# Emergence of the Novel Aminoglycoside Acetyltransferase Variant *aac(6′)-Ib-D179Y* and Acquisition of Colistin Heteroresistance in Carbapenem-Resistant *Klebsiella pneumoniae* Due to a Disrupting Mutation in the DNA Repair Enzyme MutS

**DOI:** 10.1128/mBio.01954-20

**Published:** 2020-12-22

**Authors:** Toyotaka Sato, Takayuki Wada, Suguru Nishijima, Yukari Fukushima, Chie Nakajima, Yasuhiko Suzuki, Satoshi Takahashi, Shin-ichi Yokota

**Affiliations:** a Department of Microbiology, Sapporo Medical University School of Medicine, Sapporo, Japan; b Human Life Science, Graduate School of Human Life Science, Osaka City University, Osaka, Japan; c Graduate School of Advanced Science and Engineering, Waseda University, Tokyo, Japan; d Computational Bio-Big Data Open Innovation Laboratory, National Institute of Advanced Industrial Science and Technology, Tokyo, Japan; e Structural and Computational Biology Unit, European Molecular Biology Laboratory, Heidelberg, Germany; f Division of Bioresources, Hokkaido University Research Center for Zoonosis Control, Sapporo, Japan; g Global Station for Zoonosis Control, Global Institution for Collaborative Research and Education (GI-CoRE), Hokkaido University, Sapporo, Japan; h Division of Laboratory Medicine, Sapporo Medical University Hospital, Sapporo, Japan; i Department of Infection Control and Laboratory Medicine, Sapporo Medical University School of Medicine, Sapporo, Japan; Louis Stokes Veterans Affairs Medical Center

**Keywords:** antimicrobial resistance, *mutS*, *qnrB91*, *aac(6′)-Ib-D179Y*, amikacin resistance, colistin resistance, *Klebsiella pneumoniae*

## Abstract

Amikacin and colistin are effective against carbapenem-resistant Klebsiella pneumoniae. In 2017, we successively isolated three carbapenem-resistant K. pneumoniae isolates (ST967) from a patient with chronic renal failure in Japan. The first (SMKP01, sputum, day 0) and second (SMKP02, blood, day 14) strains were resistant to most antimicrobials tested but still susceptible to amikacin (MICs of 4 and 0.5 mg/liter, respectively) and colistin (MIC of 0.5 mg/liter for both). The third strain (SMKP03, blood, day 51) was not susceptible to amikacin (MIC, 32 mg/liter), and its MIC for colistin varied (0.5 to 8 mg/liter). Whole-genome sequencing of SMKP01 revealed that 17 of 20 antimicrobial resistance genes, including *qnrB91* (a novel *qnrB2* variant) and *aac(6′)-Ib-cr*, were located on an 86.9-kb IncFII-IncQ plasmid. The *qnrB91* conferred greater fluoroquinolone resistance than *qnrB2*. SMKP03 *aac(6′)-Ib-cr* that possessed a gene mutation that resulted in an R102W substitution, namely, *aac(6′)-Ib-D179Y*, made a greater contribution to amikacin resistance than did *aac(6′)-Ib-cr*. SMKP03 harbored a nonsense mutation in *mutS*, which encodes a DNA repair enzyme. Introduction of this mutation into SMKP01 (SMKP01*mutS*_A307T_) resulted in a dramatic increase (>58-fold) in the frequency of spontaneous amikacin-resistant mutants relative to SMKP01, and the substantial mutants possessed *aac(6′)-Ib-D179Y*. SMKP01*mutS*_A307T_ exhibited an unstable MIC for colistin (0.5 to 8 mg/liter). The results demonstrate that a disruptive mutation in MutS, arising during the clinical course of an infection, created a platform for the acquisition of amikacin nonsusceptibility and colistin heteroresistance in multidrug-resistant K. pneumoniae, mediated by the elevated frequency of spontaneous mutations.

## INTRODUCTION

Klebsiella pneumoniae, an important nosocomial pathogen, can cause infections of the respiratory and urinary tracts, as well as bacteremia (1). Antimicrobial-resistant K. pneumoniae is more prone to cause such infections. Accordingly, the emergence of carbapenem-resistant K. pneumoniae is of serious clinical concern. Amikacin is the antimicrobial of choice for treating carbapenem-resistant K. pneumoniae because most isolates continue to be susceptible to amikacin (2, 3). Colistin and tigecycline are last-line drugs for fighting multidrug-resistant Gram-negative bacteria (4, 5).

Amikacin resistance in *Enterobacteriaceae* mainly occurs via the acquisition of genes encoding acetyltransferase [*aac(6′)-Ib*] and 16S rRNA methylation enzymes (*rmtA*, *rmtB*, *rmtC*, *rmtD*, and *armA*) (2). Colistin resistance in *Enterobacteriaceae* is mediated by the two-component systems PhoPQ and PmrAB (6–9). Aberrant activation, which is mainly attributed to amino acid substitutions of PhoPQ and/or PmrAB, upregulates the expression of the downstream genes *eptA* and *arnT*, which encode phosphoethanolamine transferase and 4-amino-4-deoxy-l-arabinose transferase, respectively (6). In addition, dissemination of plasmid-meditated colistin resistance (*mcr*) genes that encode phosphoethanolamine transferases, especially in *Enterobacteriaceae*, is of worldwide concern (5).

In this study, we successively isolated three carbapenem-resistant K. pneumoniae isolates during the clinical course of a patient in Japan. Although the first and second strains were susceptible to amikacin and colistin, the third strain was no longer susceptible to amikacin and exhibited unstable susceptibility to colistin (ranging from a susceptible to a resistant phenotype). We analyzed the resistance mechanisms observed in the third strain.

## RESULTS

### Isolation and antimicrobial susceptibility of three carbapenem-resistant *K. pneumoniae* isolates.

Among three carbapenem-resistant K. pneumoniae isolates obtained from the patient with chronic renal failure, the first (SMKP01) and second (SMKP02) were resistant to most antimicrobials tested but still susceptible to amikacin (MIC of 2 and 0.5 mg/liter, respectively) and colistin (MIC of 0.5 mg/liter for both) (Table 1). The third strain (SMKP03) was not susceptible to amikacin (MIC of 32 mg/liter), and the MIC for colistin was unstable (MICs ranging from 0.5 to 8 mg/liter, by multiple repetition of the susceptibility test). These three strains were the same multilocus sequence type (MLST), ST967.

**TABLE 1:** Antimicrobial susceptibility of K. pneumoniae strains SMKP01, SMKP02, and SMKP03^*a*^

Antimicrobial agent	Breakpoint (mg/liter)	MIC (mg/liter)		
		SMKP01	SMKP02	SMKP03
Piperacillin	≥128	>128 (R)	>128 (R)	>128 (R)
Sulbactam-ampicillin	≥32	>128 (R)	>128 (R)	>128 (R)
Amoxicillin-clavulanic acid	≥32	64 (R)	64 (R)	>128 (R)
Piperacillin-tazobactam	≥128	>128 (R)	>128 (R)	>128 (R)
Ceftazidime	≥16	64 (R)	>128 (R)	>128 (R)
Cefepime	≥16	>128 (R)	>128 (R)	>128 (R)
Imipenem	≥4	2 (I)	4 (R)	4 (R)
Meropenem	≥4	4 (R)	4 (R)	4 (R)
Doripenem	≥4	2 (I)	2 (I)	4 (R)
Biapenem	NA	1	2	8
Ciprofloxacin	≥1	64 (R)	16 (R)	64 (R)
Levofloxacin	≥2	64 (R)	16 (R)	64 (R)
Moxifloxacin	>0.25*	32 (R)	16 (R)	64 (R)
Sitafloxacin	NA	4	2	8
Gentamicin	≥16	>128 (R)	0.25 (S)	>128 (R)
Amikacin	≥64	2 (S)	0.5 (S)	32 (I)
Minocycline	≥16	128 (R)	128 (R)	>128 (R)
Tigecycline	>0.5*	4 (R)	8 (R)	8 (R)
Colistin	≥4	0.5 (S)	0.5 (S)	0.5–8

a NA, not available; S, susceptible; I, intermediate; R, resistant. *, According to EUCAST Clinical Breakpoint 2020.

### Identification of antimicrobial resistance genes, and OmpK35 and OmpK36 profiles, of three carbapenem-resistant *K. pneumoniae* isolates.

Whole-genome sequencing revealed a total of 20 antimicrobial resistance genes. All isolates were positive for *bla*_SHV-27_, *bla*_CTX-M-3_, *aph(3′)-Ia*, *strA*, *strB*, *qnrS1*, *oqxAB*, *fosA*, *floR*, *sul2*, and *tetA*. SMKP01 and SMKP03, but not SMKP02, were positive for *bla*_TEM-1B_, *aac(6′)-Ib-cr*, *aac(3)-IId*, *aadA16*, *qnrB2-like*, *mphA*, *ARR-3*, *sul1*, and *dfrA27*. None of the known genes encoding carbapenemases were detected in any of the three strains.

We identified a novel *qnrB2-like* variant with the R87C amino acid substitution due to a single-nucleotide mutation at nucleotide position 259 (CGC→GGC). We registered this variant as *qnrB91* according to the recommendation of Jacoby et al. (https://externalwebapps.lahey.org/studies/QNR_Studies.aspx) (10).

**FIG 1:**
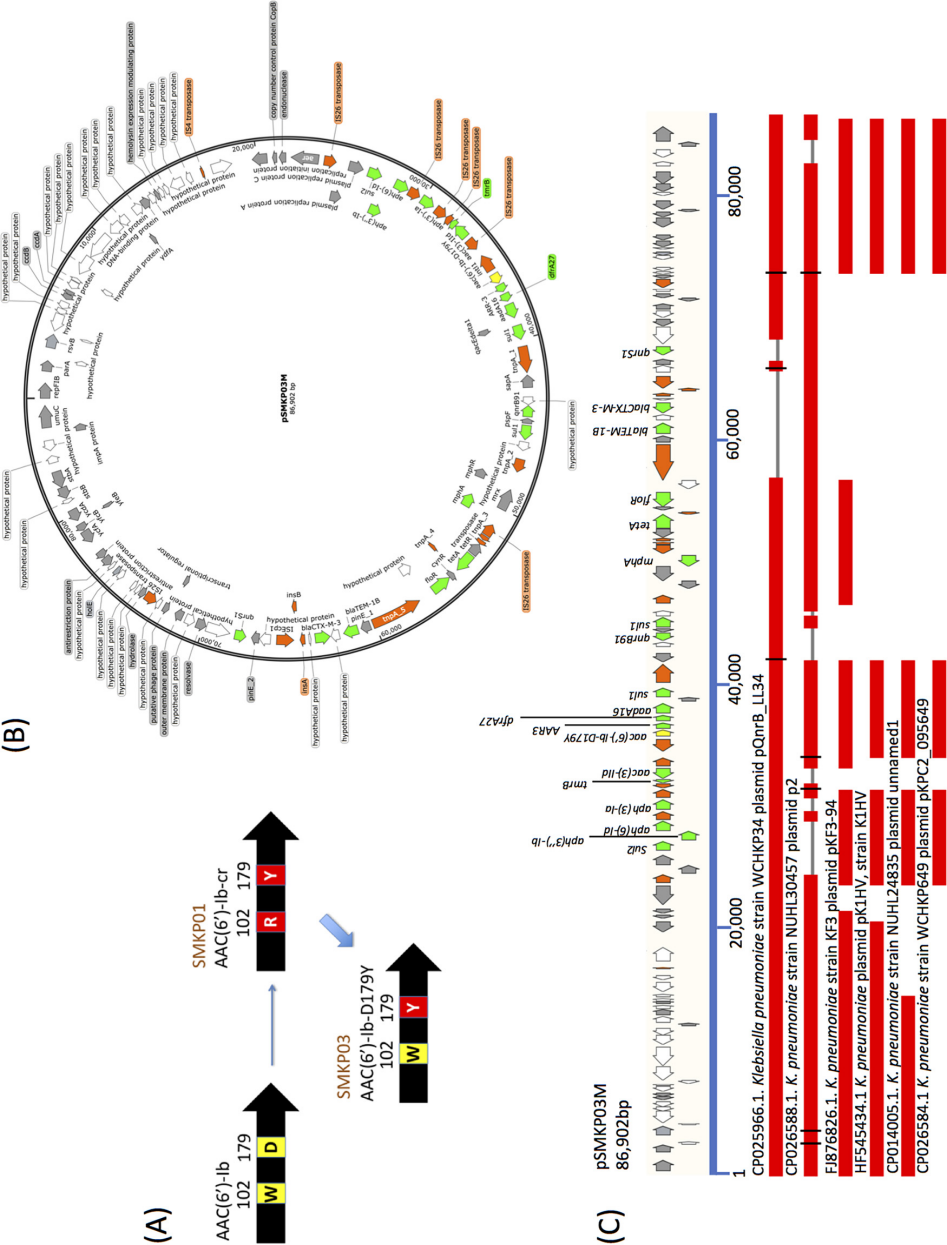
Presence of *aac(6′)-Ib-D179Y* in SMKP03, genomic analysis of pSMKP03, and comparison with other plasmids by BLAST search. (A) Occurrence of AAC(6′)-Ib-D179Y in SMKP03. AAC(6′)-Ib-D179Y harbored an amino acid substitution, R102W, relative to AAC(6′)-Ib-cr in SMKP01; thus, AAC(6′)-Ib-D179Y is genetically intermediate between AAC(6′)-Ib and AAC*(6′)*-Ib-cr due to possession of chimeric amino acid substitutions 102W and 179Y. (B) Genomic analysis of pSMKP03. (C) BLAST search for comparison with similar plasmids. Colored arrows indicate CDSs: antimicrobial resistance genes (green), *aac(6′)-Ib-D179Y* (yellow), mobile gene elements (orange), genes encoding functional proteins (white), and genes encoding hypothetical proteins (gray).

SMKP03 harbored a novel variant of *aac(6′)-Ib-cr*, *aac(6′)-Ib-D179Y*. The single-nucleotide mutation at nucleotide position 304 (CGG→TGG) corresponds to the amino acid substitution R102W. Thus, the mutation represented a chimeric variant of AAC(6′)-Ib-cr and AAC(6′)-Ib (Fig. 1A).

Three antimicrobial resistance genes (*bla*_SHV-27_, *oqxAB*, and *fosA*) were located on the chromosome. These resistance genes have been known to be intrinsic resistance in K. pneumoniae (11–13). The *oqxB* mRNA expression level of SMKP02 was lower than those of SMKP01 and SMKP03 (*P* < 0.01). *bla*_SHV-27_ and *fosA* mRNA expressions were not drastically changed among the strains (see Fig. S1 in the supplemental material). The 17 other antimicrobial resistance genes were located on an 86.9-kb IncFII-IncQ1 plasmid (Fig. 1B). In SMKP02, the plasmid lacked 10 of the antimicrobial resistance genes [*bla*_TEM-1B_, *qnrB91*, *aac(6′)-Ib-cr*, *aph(3′)-Ia*, *aac(3′)-IId*, *aadA16*, *mph*(*A*), *AAR-3*, *sul1*, and *dfrA27*]. In SDS-PAGE analysis, none of the bands corresponding to OmpK35 or OmpK36 were observed in any of the three strains (see Fig. S2). The *acrB* mRNA expression level was more than 2-fold higher in SMKP01, SMKP02, and SMKP03 than in K. pneumoniae ATCC 13383 (see Fig. S3). In Resfinder analysis, we detected several nonsynonymous mutations in *acrR* (P161R, G164A, F172S, R173G, L195V, F197I, and K201M) in the three strains. The *acrB* expression was the highest in SMKP03 among the strains (*P* < 0.05) (see Fig. S1).

10.1128/mBio.01954-20.1 FIG S1 mRNA expression levels of chromosomal antimicrobial resistance genes, *acrB*, *oqxB*, *bla*_SHV-27_, and *fosA* in SMKP01, SMKP02, and SMKP03. Values on the *y* axis are the relative expression levels (fold change) normalized against levels in SMKP01 as 1.0. * and **, significant differences from SMKP01 (*P* < 0.05 and *P* < 0.01, respectively). Download FIG S1, PDF file, 0.1 MB. Copyright © 2020 Sato et al. 2020 Sato et al. This content is distributed under the terms of the Creative Commons Attribution 4.0 International license.

10.1128/mBio.01954-20.2 FIG S2 Omp profiling of SMKP01, SMKP02, and SMKP03. Lane 1, ATCC 13883; lane 2, SMKP01; lane 3, SMKP02; lane 4; SMKP03. Download FIG S2, PDF file, 0.2 MB. Copyright © 2020 Sato et al. 2020 Sato et al. This content is distributed under the terms of the Creative Commons Attribution 4.0 International license.

10.1128/mBio.01954-20.3 FIG S3 Relative *acrB* mRNA expression levels (versus K. pneumoniae ATCC 13383) in SMKP01, SMKP02, and SMKP03. Values on the *y* axis are the relative expression levels (fold change) normalized against levels in ATCC 13383 as 1.0. * and **, significant differences from SMKP01 (*P* < 0.05 and *P* < 0.01, respectively). Download FIG S3, PDF file, 0.04 MB. Copyright © 2020 Sato et al. 2020 Sato et al. This content is distributed under the terms of the Creative Commons Attribution 4.0 International license.

### Comparison of *qnrB91* and *qnrB2* with respect to fluoroquinolone susceptibility.

We constructed expression vectors for *qnrB91* or *qnrB2.* Introduction of either vector into K. pneumoniae ATCC 13883 increased the fluoroquinolone MIC, although the effect of *qnrB91* was >8-fold greater than that of *qnrB2* (Table 2).

**TABLE 2:** Comparison of of *qnrB91* and *qnrB2* fluoroquinolone MICs for K. pneumoniae ATCC 13883^*a*^

Strain	QnrB2	MIC (mg/liter)^*b*^			
	Arg87	CIP	LVX	NOR	STX
ATCC 13883		0.06	0.06	0.25	0.01
ATCC 13883/pSTV28		0.06	0.06	0.125	0.01
ATCC 13883/pSTV*qnrB2*	WT	0.5 (×8)	0.5 (×8)	1 (×8)	0.25 (×16)
ATCC 13883/pSTV*qnrB91*	Cys	4 (×64)	4 (×64)	8 (×64)	2 (×128)

a CIP, ciprofloxacin; LVX, levofloxacin; NOR, norfloxacin; STX, sitafloxacin; WT, wild type.

b The fold difference versus ATCC 13883/pSTV28 is indicated in parentheses.

### Comparison of *aac(6′)-Ib-cr* and *aac(6′)-Ib-D179Y* with respect to susceptibilities to amikacin and fluoroquinolones.

We next evaluated the contribution of *aac(6′)-Ib-cr* in SMKP01 and *aac(6′)-Ib-D179Y* in SMKP03 to amikacin and fluoroquinolone susceptibilities. To this end, we introduced expression vectors for a series of *aac(6′)-Ib* genes [*aac(6′)-Ib* (102W, 179D), *aac(6′)-Ib-cr* (102R, 179Y), *aac(6′)-Ib-D179Y* (102W, 179Y), and *aac(6′)-Ib-W102R* (102R, 179D)] into K. pneumoniae ATCC 13883 (Table 3). Expression of *aac(6′)-Ib* yielded the highest amikacin MIC, followed by *aac(6′)-Ib-D179Y*, *aac(6′)-Ib-W102R*, and *aac(6′)-Ib-cr.* The MICs for ciprofloxacin and norfloxacin were the highest in cells expressing *aac(6′)-Ib-cr*, followed by *aac(6′)-Ib-D179Y*, *aac(6′)-Ib*, and *aac(6′)-Ib-W102R.*

**TABLE 3:** Comparison of fluoroquinolone and aminoglycoside MICs of *aac(6′)-Ib-D179Y* and other *aac(6′)-Ib* variants in K. pneumoniae ATCC 13883^*a*^

Strain	*aac(6′)*-*Ib*		MIC (mg/liter)^*b*^					
	Trp102	Asp179	CIP	LVX	NOR	STX	GEN	AMK
ATCC 13883			0.06	0.06	0.25	0.01	<0.25	0.5
ATCC 13883/pSTV28			0.06	0.06	0.25	0.01	<0.25	0.5
ATCC 13883/pSTV*aac(6′)-Ib*	WT	WT	0.06	0.06	0.25	0.01	0.5 (×>2)	8 (×16)
ATCC 13883/pSTV*aac(6′)-Ib-cr*	Arg	Tyr	0.125 (×2)	0.06	1 (×4)	0.01	0.5 (×>2)	1 (×2)
ATCC 13883/pSTV*aac(6′)-Ib-D179Y*	WT	Tyr	0.125 (×2)	0.06	0.5 (×2)	0.01	0.5 (×>2)	4 (×8)
ATCC 13883/pSTV*aac(6′)-Ib-W102R*	Arg	WT	0.06	0.06	0.25	0.01	0.5 (×>2)	2 (×4)

a CIP, ciprofloxacin; LVX, levofloxacin; NOR, norfloxacin; STX, sitafloxacin; GEN, gentamicin; AMK, amikacin; WT, wild type.

b The fold difference versus ATCC 13883/pSTV28 is indicated in parentheses.

### Numbers of single-nucleotide polymorphisms in SMKP02 and SMKP03 relative to SMKP01.

**FIG 2:**
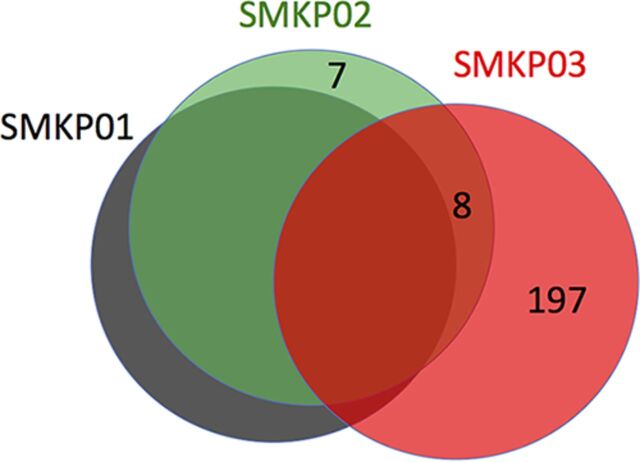
Comparison of numbers of SNPs and mutagenesis analysis. The numbers of SNPs in SMKP02 and SMKP03 are shown relative to SMKP01.

According to our calculation of accumulated mutations based on whole-genome sequences, SMKP02 and SMKP03 had 15 and 205 single-nucleotide polymorphisms (SNPs), respectively, relative to SMKP01 (Fig. 2; see also Tables S1 and S2). SMKP03, which accumulated many more SNPs, harbored a nonsense mutation (A307T) in the DNA repair gene *mutS*, which introduced a stop codon (AAG→TAG) at amino acid position 103 of MutS (see Table S2). Neither SMKP01 nor SMKP02 harbored this mutation (see Table S1).

10.1128/mBio.01954-20.5 TABLE S1 Single-nucleotide polymorphisms detected in SMKP02 compared to SMKP01. Download Table S1, XLSX file, 0.01 MB. Copyright © 2020 Sato et al. 2020 Sato et al. This content is distributed under the terms of the Creative Commons Attribution 4.0 International license.

10.1128/mBio.01954-20.6 TABLE S2 Single-nucleotide polymorphisms detected in SMKP03 compared to SMKP01. Download Table S2, XLSX file, 0.03 MB. Copyright © 2020 Sato et al. 2020 Sato et al. This content is distributed under the terms of the Creative Commons Attribution 4.0 International license.

### Determination of spontaneous mutation frequency using mitomycin C.

**FIG 3:**
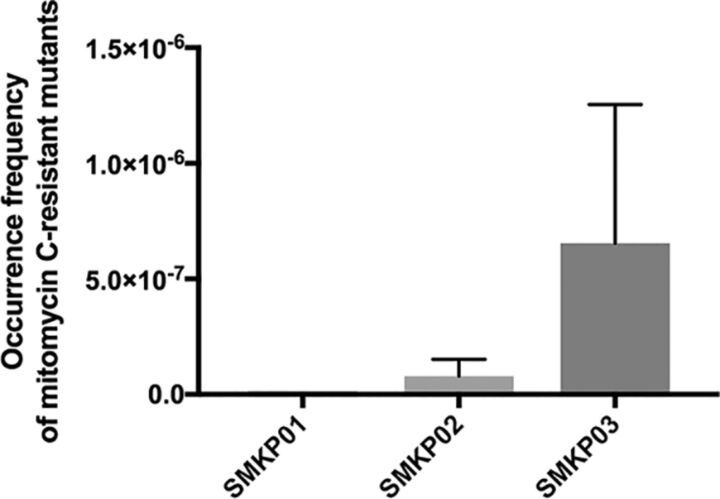
Frequency of mitomycin C-resistant mutants of SMKP01, SMKP02, and SMKP03. Spontaneous mitomycin C-resistant mutants were selected on LB agar containing 8 mg/liter of mitomycin C after cultivation for 48 h at 37°C.

To evaluate gene mutation frequency, we measured the number of spontaneous mutants that grew on Luria-Bertani (LB) agar containing mitomycin C (Fig. 3). The frequencies of spontaneous mutations were 2.1 × 10^−9^ and 7.8 × 10^−8^ in SMKP01 and SMKP02, respectively (*P* = 0.359). In SMKP03, the frequency (6.5 × 10^−7^) was 314-fold higher than in SMKP01 (*P* = 0.051).

### Frequencies of amikacin-resistant mutants arising from SMKP01 harboring a mutation in *mutS*.

**FIG 4:**
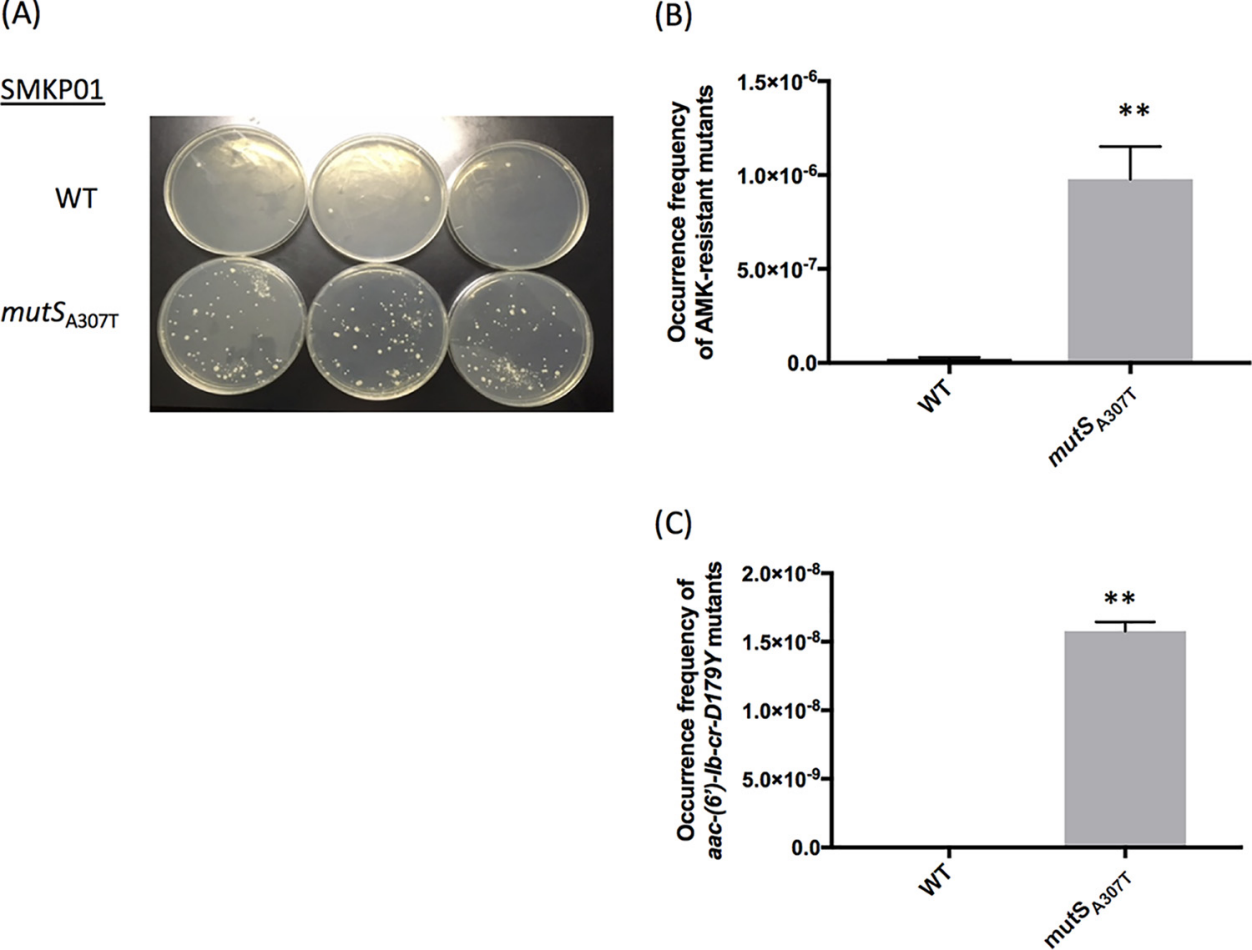
Influence of nonsense *mutS* mutation upon acquisition of amikacin resistance in SMKP01. (A) Spontaneous amikacin-resistant mutants selected on Mueller-Hinton II agar containing 16 mg/liter of amikacin after cultivation for 48 h at 37°C. (B) Frequency of spontaneous amikacin-resistant mutants. (C) Frequency of amikacin-resistant mutants possessing *aac(6′)-Ib-D179Y*. AMK, amikacin. **, *P* < 0.01.

We used pORTMAGE to construct a SMKP01 derivative harboring the *mutS* mutation from SMKP03, SMKP01*mutS*_A307T_. SMKP01*mutS*_A307T_ had a higher frequency of spontaneous amikacin-resistant mutants (9.8 × 10^−7^), as determined by selection with amikacin *in vitro*, relative to the parental strain (1.7 × 10^−8^) (57.7-fold; *P* < 0.01) (Fig. 4A and B). The R102W mutation in AAC(6′)-Ib-cr [AAC(6′)-Ib-D179Y], which was detected in SMKP03, was only observed in selected mutants derived from SMKP01*mutS*_A307T_ (Fig. 4C). These results suggested that the nonsusceptibility of SMKP03 to amikacin was acquired due to the R102W point mutation in AAC(6′)-Ib-cr and that this mutation arose due to the disruption of MutS.

### Occurrence of colistin-resistant mutants in SMKP03.

**FIG 5:**
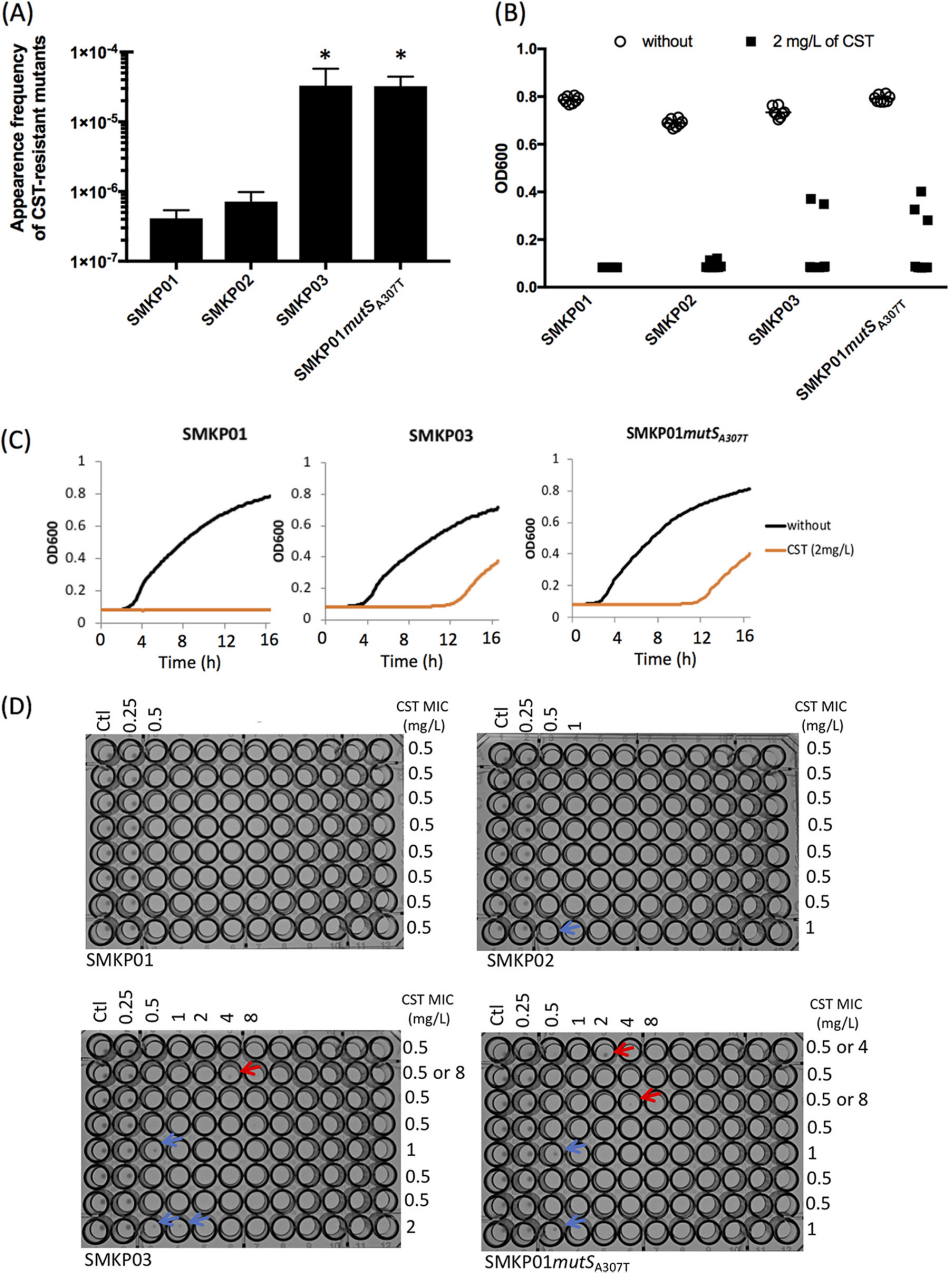
Influence of nonsense *mutS* mutation on acquisition of colistin resistance. (A) Frequency of spontaneous amikacin-resistant mutants. *, *P* < 0.05. (B) Growth of K. pneumoniae strains in the presence or absence of colistin (2 mg/liter). Each well contained 100 μl of Mueller-Hinton II broth and was inoculated with 1 × 10^5^ CFU/ml of K. pneumoniae. Bacterial growth was measured by determining the OD_600_ after cultivation for 16 h at 37°C. Each condition was evaluated eight times independently. (C) Growth curves of SMKP01, SMKP03, and SMKP01*mutS*_A307T_. Figures are representative of eight individual examinations. (D) Colistin susceptibility test of SMKP01, SMKP02, SMKP03, and SMKP01*mutS*_A307T_. The test was performed in octuplicate, and the MIC was determined in each assay. Red arrows indicate bacterial growth at a colistin concentration beyond the colistin breakpoint (>2 mg/liter). Blue arrows indicate bacterial growth at a higher colistin concentration than SMKP01 (>0.5 mg/liter). CST, colistin; Ctl, control.

SMKP03 exhibited an unstable MIC for colistin that varied among examinations (Table 1). In the MIC measurement, “skip-well” phenomena were observed in SMKP03 but not in SMKP01 and SMKP02 (Fig. 5D). Hence, we examined the emergence of colistin-resistant mutants from these strains and SMKP01*mutS*_A307T_ in the presence of colistin (2 mg/liter).

SMKP03 and SMKP01*mutS*_A307T_ had a markedly higher frequency of colistin-resistant colonies (3.3 × 10^−5^ and 3.2 × 10^−5^, respectively) than SMKP01 (4.1 × 10^−7^) (80.0-fold [*P* = 0.02] and 78.8-fold [*P* = 0.01], respectively) (Fig. 5A). In the broth culture, the colistin-resistant mutants were emerged in the presence of 2 mg/liter colistin in several culture batches of SMKP03 and SMKP01*mutS*_A307T_ but not in all batches of SMKP01 and SMKP02 (Fig. 5B and C). The colistin-resistant mutants derived from SMKP01*mutS*_A307T_ had colistin MICs from 16 to >64 mg/liter, and these values did not alter after several passages in plain Mueller-Hinton II broth.

To investigate the mechanism underlying the emergence of colistin resistance and the unstable MIC in SMKP03, we examined the whole-genome sequences of three colistin-resistant mutants derived from SMKP03 (SMKP03CST-1, SMKP03CST-2, and SMKP03CST-3), which revealed 45, 65, and 63 nonsynonymous mutations, respectively (see Table S3). SMKP03CST-1 harbored an amino acid substitution in PhoP (Glu82Leu) (see Table S1). SMKP03CST-2, and SMKP03CST-3 harbored an amino acid substitution in PmrB (Ser203Pro) (see Table S3). In population analysis profile (PAP) analysis, these colistin-resistant mutants exhibited stable colistin resistance phenotype, although SMKP03 was defined as colistin-heteroresistant phenotype (see Fig. S4).

10.1128/mBio.01954-20.7 TABLE S3 Nonsynonymous mutations occurred in SMKP03-derived colistin-resistant mutants, SMKP03CST-1 (Table S3-1), SMKP03CST-2 (Table S3-2), and SMKP03CST-3 (Table S3-3). Download Table S3, XLSX file, 0.03 MB. Copyright © 2020 Sato et al. 2020 Sato et al. This content is distributed under the terms of the Creative Commons Attribution 4.0 International license.

10.1128/mBio.01954-20.8 TABLE S4 Detection of nonsynonymous mutations in *mutS* in K. pneumoniae isolates from the BLASTn database. Red letters show putative disrupted mutations. Download Table S4, XLSX file, 0.04 MB. Copyright © 2020 Sato et al. 2020 Sato et al. This content is distributed under the terms of the Creative Commons Attribution 4.0 International license.

### Prevalence of *K. pneumoniae mutS*-null isolates.

To evaluate the dissemination of K. pneumoniae isolates with putative disruptions of functional MutS, we sought to identify *mutS*-null mutations (such as nonsense mutations, frameshift mutations, gene deletions, and gene insertions) from a database of complete genome sequences of 454 K. pneumoniae isolates. A total of 3.1% (14/454 isolates) of K. pneumoniae isolates possessed the *mutS*-null mutation. These 14 isolates were composed of different MLSTs (see Table S4).

## DISCUSSION

**FIG 6:**
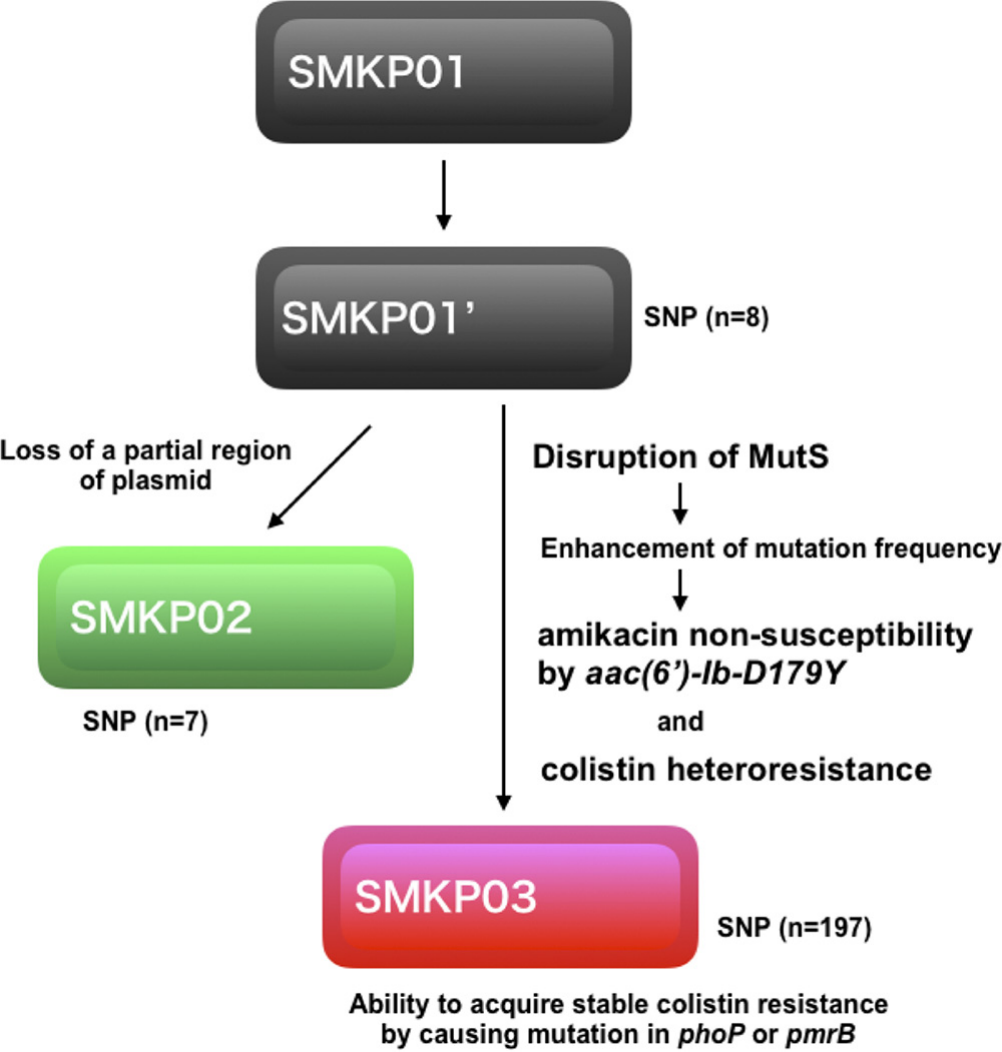
Summary of emergence of SMKP02 and SMKP03 from SMKP01 during course of infections in a patient. First, K. pneumoniae SMKP01, which caused respiratory tract infection, was isolated from the sputum of the patient. Second, SMKP01 (SMKP01’) lost a part, including 10 of the antimicrobial resistance genes [*bla*_TEM-1B_, *qnrB91*, *aac(6′)-Ib-cr*, *aph*(*3′*)*-Ia*, *aac*(*3′*)*-IId*, *aadA16*, *mph*(*A*), *AAR-3*, *sul1*, and *dfrA27*], of the plasmid and invaded into the blood (SMKP02). Third, SMKP01 (SMKP01’) caused a nonsense mutation (A307T) in the DNA repair gene *mutS*, which introduced a stop codon (AAG→TAG) at amino acid position 103 of MutS, and invaded into the blood (SMKP03). Due to the disruption of MutS function, SMKP03 accumulated spontaneous mutations, including a single-nucleotide mutation at nucleotide position 304 (CGG→TGG), that correspond to the amino acid substitution R102W in *aac(6′)-Ib-cr*, namely, *aac(6′)-Ib-D179Y*. SMKP03 also exhibited a heteroresistant phenotype to colistin and acquired the ability to evoke stable colistin resistance via mutations in *phoP* or *pmrB*. SNP, single-nucleotide polymorphism.

In this study, we observed *mutS*-mediated acquisition of resistance (or nonsusceptibility) to amikacin and colistin in K. pneumoniae during the clinical course in a patient (Fig. 6). MutS is a DNA mismatch repair enzyme that is essential for combating the adverse effects of damage on the genome. Functional disruption of MutS by frameshift or gene deletion leads to a hypermutable phenotype (14, 15). Six regions of MutS, N-1′ (FVP, 567–569), N-1 (GPNMAGKS, 583–590), N-3 (DE, 662–663), N-3′ (TH, 695–696), and N-4 (H726) of subunit A and the N-2 region (ST, 637–638) of subunit B are required for ATPase activity, which plays a proofreading role in DNA mismatch repair in E. coli (16). These functional regions were truncated by a nonsense mutation in MutS of K. pneumoniae isolate SMKP03, and the strain exhibited a hypermutable phenotype.

The hypermutable phenotype acquired by disruption of MutS confers a selective advantage despite the fact that inactive MutS is inferior to the wild type in a nonselective environment (15). Consistent with this, SMKP03 developed amikacin nonsusceptibility and colistin resistance during exposure to those agents *in vitro* more frequently than the two other isolates, SMKP01 and SMKP02, which lacked mutations in MutS. These observations suggested that acquisition of a hypermutable phenotype caused by loss of MutS function was an adaptive strategy for overcoming antimicrobial selection pressure. In other words, functional disruption of MutS offers a platform for acquisition of novel resistance mechanisms via novel mutations. *aac(6′)-Ib-D179Y* is a previously unreported chimeric variant of *aac(6′)-Ib* and *aac(6′)-Ib*-*cr* (Fig. 1). AAC(6′)-Ib-cr is a variant of aminoglycoside acetyltransferase AAC(6′)-Ib, differing by two amino acid substitutions, W102R and D179Y (17). These substitutions confer N-acetylation at the amino group piperazine ring of several fluoroquinolones (17), such as ciprofloxacin and norfloxacin, thereby increasing resistance to these compounds; in contrast, they provide a lower level of amikacin resistance than AAC(6′)-Ib (Table 3). We found that the novel variant, AAC(6′)-Ib-D179Y, provided levels of amikacin and fluoroquinolone resistance comparable to those conferred by AAC(6′)-Ib and AAC(6′)-Ib-cr, respectively (Table 3). The patient had been treated with amikacin (from day 15 to day 70), suggesting that AAC(6′)-Ib-D179Y was generated by spontaneous mutation in a MutS-disrupted strain SMKP03 and subsequently selected during amikacin treatment.

The instability of the colistin MIC in SMKP03 was also due to disruption of functional MutS. MutS disruption dramatically increased the frequency of colistin-resistant mutants and led to “skip-well” phenomena in MIC tests of colistin (Fig. 5). The frequency of such mutants in SMKP03 was 2.5 × 10^−5^, suggesting that spontaneous colistin-resistant mutants might have sometimes emerged during the MIC tests. Indeed, PAP test demonstrated that SMKP03 exhibited colistin-heteroresistant phenotype. Although previous studies reported the contribution of *pmrAB* or *phoPQ* mutation to colistin heteroresistance in K. pneumoniae (18, 19), the identity of the bacterial factor(s) that generate this mutation was still unclear. This study first demonstrated that the *mutS* mutation contributes this mechanism. These might cause difficulty with colistin susceptibility testing in clinical laboratories. The patient had no clinical history of colistin treatment. Frequent emergence of spontaneous colistin resistance and the ability to evoke stable colistin resistance by a mutation occurring in *phoP* or *pmrB* in multidrug-resistant K. pneumoniae has a clinical impact because colistin is a last-line drug for treatment of this type of infection. Thus, our observations are a reminder that colistin administration during the course of an infection can easily select for colistin-resistant K. pneumoniae possessing an *mutS* mutation.

In this case, none of the genes encoding carbapenemases were detected in any of the three strains. Resistant and intermediate phenotypes to imipenem, meropenem, and doripenem may be explained by the combination of β-lactamase, CTX-M-3, and the absence of porins, OmpK35, and OmpK36, in K. pneumoniae, as seen in the present study and in previous studies (20, 21). We also detected mutations of genes that encode multidrug efflux pumps (such as *mdtA*, *mdtM*, *acrB*, and *oqxAB*) in SMKP03 (see Table S2). Among them, the mRNA expression of *acrB* was higher in SMKP03 than in SMKP01 and SMKP02 (*P* < 0.05). AcrAB is a major multidrug efflux pump that excretes several antimicrobials such as fluoroquinolones, minocycline, and tigecycline (22–24). The expression levels were regulated by SoxS, MarR, AcrR, and RamR (25–27). Thus, these mutations may contribute to the enhancement of moxifloxacin, sitafloxacin, and minocycline MICs in SMKP03, since *soxS* mutation was detected only in SMKP03 (see Table S2). SMKP01, SMKP02, and SMKP03 exhibited resistance to tigecycline. This may be explained by the higher *acrB* expression levels in SMKP01, SMKP02, and SMKP03 than that in the tigecycline-susceptible strain, ATCC 13383, due to several nonsynonymous mutations in *acrR* (P161R, G164A, F172S, R173G, L195V, F197I, and K201M) and RamR (T43A, K194Stop). Tigecycline is another option against multidrug-resistant Gram-negative bacteria with colistin. Therefore, the acquisition of colistin resistance by multidrug-resistant K. pneumoniae isolates with tigecycline resistance (as seen in SMKP03) is a serious problem in a clinical setting.

SMKP01 and SMKP03 carried a plasmid that harbored numerous resistance genes against multiple classes of antimicrobial agents. A BLAST search suggested that this plasmid was generated through combination of two plasmids, pQnrB_LL34 and p2, both previously reported in K. pneumoniae. This observation alerts us to the emergence of a plasmid capable of conferring a high degree of multidrug resistance. Among the resistance genes located on this plasmid, we identified a novel *qnrB* variant, *qnrB91*, which conferred a higher level of resistance to fluoroquinolones than *qnrB2*. Dissemination of such a plasmid, which causes the development and accumulation of multidrug resistance, among *Enterobacteriaceae* clinical isolates could make it more difficult to choose effective antimicrobial agents to combat infections in the future.

In conclusion, our results demonstrate that MutS disruption that arose during the clinical course in a patient led to the acquisition of amikacin nonsusceptibility and colistin resistance in multidrug-resistant K. pneumoniae. A previous study reported the frequent isolation of hypermutable Haemophilus influenzae with mutations in *mutS* from cystic fibrosis patients, as well as the clinical advantages of these mutants (28). Given that a substantial fraction of K. pneumoniae isolates (3.1% of entire K. pneumoniae isolates available in BLASTn search) harbor *mutS*-null mutations, these mutations probably affect the clinical condition, the effectiveness of antimicrobial treatment, and the diagnosis of antimicrobial susceptibilities. Therefore, *mutS* mutation should be taken into account when considering novel emergence mechanisms associated with bacterial survival and antimicrobial resistance in clinical settings.

## MATERIALS AND METHODS

### Bacterial isolation and antimicrobial susceptibility testing.

We successively isolated three carbapenem-resistant K. pneumoniae isolates from a patient with chronic renal failure at Sapporo Medical University Hospital in 2017. The first strain (SMKP01) was isolated from sputum at day 0. The second (SMKP02) and third strains (SMKP03) were isolated blood at day 13 and day 50, respectively. Susceptibility to antimicrobials was determined by measuring MICs according to CLSI guidelines (29). This study was approved by Sapporo Medical University School of Medicine Ethics Committee (no. 302-1031).

### Construction of expression vectors for *qnrB91* and *aac-(6′)-Ib-D179Y*.

Expression vectors harboring *qnrB2* (pSTV*qnrB2*), *qnrB91* (pSTV*qnrB91*), *aac(6′)-Ib-cr* [pSTV *aac(6′)-Ib-cr*], or *aac(6′)-Ib-D179Y* [pSTV *aac(6′)-Ib-D179Y*] derived from SMKP01 or SMKP03 were constructed using the low-copy-number plasmid pSTV28 (obtained from the National BioResource Project, Mishima, Japan). Briefly, (i) DNA fragments of *qnrB91* (from −311 bp upstream) and *aac(6′)-Ib-cr* (from −563 bp upstream) from SMKP01, as well as *aac(6′)-Ib-D179Y* (from −563 bp upstream) from SMKP03, were prepared by PCR using two pairs of primers, [pSTVqnrB91-F/pSTVqnrB91-R] and [pSTVaac(6′)-Ib-cr-or-D179Y-F/pSTVaac(6′)-Ib-cr-or-D179Y-R] (see Table S5). (ii) EcoRI- and HindIII-digested pSTV28 was amplified by inverse PCR using the forward primer pMWpSTVInv-qnrB (for pSTVqnrB91) or the forward primer pMWpSTVInv-AAC [for pSTVaac(6′)-Ib-D179Y] and the common reverse primer pSTV28HindIIIinvert (see Table S5). Fragments i and ii were ligated using NEBuilder HiFi DNA Assembly master mix (New England BioLabs Japan, Tokyo, Japan) and chemically transformed into E. coli DH5α. Transformants were selected on LB agar containing 30 mg/liter chloramphenicol.

pSTV*qnrB2* was constructed by the synthesis of a *qnrB2* DNA oligonucleotide (NCBI accession number, DQ351242.1) containing 311 bp of the promoter region, which was identical to the promoter region of *qnrB91* of SMKP01 and SMKP03, and 15 bp of overlapping nucleotide sequences with each 5′ (XbaI site) or 3′ (HindIII site) end sequence of pSTV28 by generating inverse PCR using the primers pMWpSTVInv-qnrB and pSTV28HindIIIinvert, followed by DpnI digestion (see Table S5). These two fragments were ligated using NEBuilder HiFi DNA Assembly master mix.

pSTV*aac(6′)-Ib* and pSTV*aac(6′)-Ib-W102R* were constructed by site-directed PCR using the primers AAC-IbWT-siteF and AAC-IbWT-siteR (see Table S5) and pSTV*aac(6′)-Ib-D179Y* or pSTV*aac(6′)-Ib-cr* as the template, followed by DpnI digestion.

10.1128/mBio.01954-20.9 TABLE S5 Primers used in this study. Boldfacing, overlapped sequence for NEB assembly; underlining, introduced codon by site-directed PCR. Download Table S5, XLSX file, 0.01 MB. Copyright © 2020 Sato et al. 2020 Sato et al. This content is distributed under the terms of the Creative Commons Attribution 4.0 International license.

After the sequence of each expression vector was confirmed, the constructs were electroporated into K. pneumoniae ATCC 13883. Three clones harboring each vector were selected and used to determine aminoglycoside and/or fluoroquinolone MICs in triplicate.

### Construction of *mutS-*mutated SMKP01 (SMKP01*mutS*_A307T_).

The nonsense mutation of *mutS* (A307T) found in SMKP03 was introduced into SMKP01 by using pORTMAGE, as described previously (30). Hygromycin-integrated pORTMAGE (pORTMAGE-hyg) was generated by replacement of *ampR* of pORTMAGE-2 (Addgene, Watertown, MA) with a hygromycin resistance cassette (Gene Bridges, Heidelberg, Germany). *ampR*-deleted pORTMAGE2 was amplified by inverse PCR using primers PORTinvF and PORTinvR (see Table S5). The hygromycin resistance cassette with 15-bp regions overlapping the 5′ and 3′ sequences of the *ampR*-deleted region of pPORTMAGE2 was amplified using the primers PORTHyg-F and PORTHyg-R. These two fragments were assembled using NEBuilder HiFi DNA Assembly master mix according to the manufacturer’s instructions and chemically transformed into E. coli DH5α. Transformants were selected on LB agar containing 100 mg/liter of hygromycin. Then, pORTMAGE-Hyg was introduced into SMKP01 by electroporation. Oligonucleotides (90 bp) for *mutS* containing A307T were designed using the Mage Oligo Design Tool (MODEST [http://modest.biosustain.dtu.dk]; MAGEmutS, ACTGGTCAATCAGGGCGAGTCGGTGGCCATTTGCGAGCAGATTGGCGACCCGGCCACCACC**t**AGGGGCCGGTCGAGCGTAAAGTGGTGCG; the underlining indicates the stop codon introduced into SMKP01; bold lowercase “**t**” indicates the introduced nucleotide). Gene replacement in the mutants was confirmed by direct DNA sequencing.

### Expression profile of OmpK35 and OmpK36.

Outer membrane proteins of SMKP01, SMKP02, SMKP03, and ATCC 13833 were prepared as described previously (31). Protein concentrations were determined by using a BCA protein assay kit (Thermo Fisher Scientific K.K., Tokyo, Japan). Portions (2 μg) of total proteins were boiled for 5 min in Laemmli sample buffer and loaded onto 12% polyacrylamide gels. The gels were then stained with Coomassie brilliant blue.

### Measurement of *acrB*, *oqxB*, *bla*_SHV-27_, and *fosA* mRNA expression by real-time reverse-transcription PCR.

SMKP01, SMKP02, SMKP03, and ATCC 13383 were cultivated in 0.5 ml of tryptic soy broth overnight. Then, 150-μl portions of the cultures were inoculated into 10 ml of Muller-Hinton II broth (cation adjusted; Becton Dickinson, Franklin, Lakes, NJ), and the cells were grown for 1.5 h at 37°C. RNA was isolated using an RNeasy Plus minikit (Qiagen, Hilden, Germany) according to the manufacturer’s instructions. The concentration of RNA was measured spectrophotometrically using an Infinite M200 PRO instrument (Tecan, Kawasaki, Japan), and 0.5 μg was used to synthesize cDNA using ReverTra Ace qPCR RT Master Mix with gDNA remover (Toyobo, Tokyo, Japan). The expression of genes encoding *acrB*, *oqxB*, *bla*_SHV-27_, and *fosA* was estimated using KOD SYBR qPCR mix (Toyobo). The primer pairs for *acrB* (acrB-F and acrB-R) (32) and *oqxB* (oqxB1806F and oqxB1806) (33) were used in previous studies. The primer pairs for *bla*_SHV-27_ and *fosA* are listed in Table S5 in the supplemental material. The PCR cycling conditions were as follows: initial activation at 95°C for 5 min, followed by 40 cycles at 95°C for 10 s and 60°C for 30 s. Reactions were performed in a LightCycler 480 II (Roche, Mannheim, Germany). *rpoB* genes served as an endogenous reference for normalizing expression levels (33). Data were calibrated against the baseline expression level of ATCC 13383, and fold changes in expression were calculated using the comparative *C_T_* method. The data are expressed as means ± the standard deviations from three independent experiments.

### Whole-genome sequencing.

Genomic DNA was isolated by using a Wizard Genomic DNA purification kit (Promega, Madison, WI). The complete genome sequence was determined using the PacBio RS SMRT Portal (Pacific Biosciences, Menlo Park, CA). The filtered subreads (in fastq format) were assembled with 300-bp paired-end reads obtained by MiSeq sequencing (Illumina, San Diego, CA) using Unicycler (version 0.4.3) (34). This yielded a large circular contig (∼5.15 Mb) and three smaller circular contigs (∼111, ∼86.9, and ∼54.5 kb). To increase the accuracy of the final sequence, the sequences were curated by mapping analysis with short reads using the CLC Genomics Workbench (Qiagen). The finalized contig lengths were 5,155,320 bp (the SMKP03 genome), 111,317 bp (pSMKP03L, the large plasmid of SMKP03), 86,902 bp (pSMKP03M, the middle plasmid of SMKP03), and 54,548 bp (pSMKP03S, the small plasmid of SMKP03). Before registration, the sequences were automatically annotated by DFAST (legacy version) based on PROKKA (35).

The SNPs of SMKP02 and SMKP03 were detected with CLC Genomic Workbench using the basic variant detection method by sorting the obtained variant using a minimum frequency of 70%. The genome sequence of SMKP01 was used as a reference. Comprehensive genetic comparison between SMKP03 and its colistin-resistant mutants was performed, and nonsynonymous mutated genes were detected by the same method used for SNP detection.

Determination of MLST and detection of antimicrobial resistance genes were performed using MLST analysis and ResFinder, respectively, at the Center for Genomic Epidemiology (http://www.genomicepidemiology.org).

### Measurement of the frequency of mitomycin C-resistant, amikacin-nonsusceptible, or colistin-resistant mutants.

Overnight cultures of strains in tryptic soy broth were spread on Mueller-Hinton II agar (Becton Dickinson)—with or without mitomycin C (8 mg/liter), amikacin (16 mg/liter), or colistin (2 mg/liter)—and incubated at 37°C for 48 h. The frequency of the mitomycin C-resistant, amikacin-nonsusceptible, or colistin-resistant mutants was calculated by dividing the number of colonies that grew on Mueller-Hinton II agar containing the corresponding agent by the number of colonies that grew on plain Mueller-Hinton II agar. Experiments were performed in triplicate.

The frequency of amikacin-resistant mutants harboring *aac(6′)-Ib-D179Y* was measured in triplicate. Appropriately 100 colonies grew on Mueller-Hinton II agar containing 16 mg/liter of amikacin; these clones were selected, and *aac(6′)-Ib-D179Y* was detected by SNP PCR (see Table S5). Sanger DNA sequencing was performed for confirmation (see Table S5).

### Bacterial growth determination.

Bacterial growth was monitored by measuring the turbidity (that is, the optical density at 600 nm [OD_600_]) using an Infinite M200 PRO multimode microplate reader (Tecan, Kawasaki, Japan). Strains (eight clones per strain) were grown in 0.5 ml of tryptic soy broth (Becton Dickinson) overnight at 37°C, and 1 × 10^5^ CFU/ml bacteria were cultured in 0.1 ml Mueller-Hinton II broth (Becton Dickinson) in a 96-well plate at 37°C with shaking at 140 rpm for 16 h. Bacterial growth curves were created based on measurements made every 10 min for 16 h at least in triplicate.

### Defining colistin heteroresistance in SMKP03 and SMKP03-derived colistin-resistant mutants.

We performed PAP test to determine whether SMKP03-derived colistin-resistant mutants exhibited heteroresistance or not according to a previous study, with slight modification (36). SMKP03 and the colistin-resistant mutants were cultured in tryptic soy broth overnight at 37°C (these strains were assessed in this test after growth at least 50 generations on Mueller-Hinton II agar without colistin). Then, 100-μl portions of an overnight culture (ca. 1 × 10^9^ CFU/ml) were spread on Mueller-Hinton II agar containing colistin (from 0.03125 to 128 mg/liter) and cultured 24 h at 37°C. The CFU were counted, and the colistin-resistant population was calculated based on the CFU that grew on Mueller-Hinton II agar containing colistin divided by the CFU that grew on Mueller-Hinton II agar without colistin. We determined “colistin heteroresistance” based on the following criteria: (i) the main population was clinically susceptible to colistin (<2 mg/liter) and (ii) the colistin concentration appearing in the colistin-resistant subpopulation (with a frequency greater than 1 × 10^−7^) was at least 8-fold greater than the highest colistin concentration that does not affect growth of the main colistin-susceptible population (37).

### Prevalence of *mutS*-null K. pneumoniae isolates in the NCBI database.

We collected the *mutS* DNA sequences of 454 complete genome sequences from K. pneumoniae isolates in the NCBI database; these represented all complete genome sequences available as of 25 April 2020. Amino acid translation from the *mutS* DNA sequence was performed using the Sequence Manipulation Suite (https://www.bioinformatics.org/sms2/translate.html). The translated sequences were aligned by MAFFT (38) to identify each nonsynonymous mutation, including likely null mutations (such as nonsense mutations, frameshifts, gene deletions, or gene insertions). K. pneumoniae SMKP01 was used as a reference. Determination of MLST was performed using MLST analysis at the Center for Genomic Epidemiology (http://www.genomicepidemiology.org) with the genome data.

### Statistical analysis.

One-way analysis of variance and Dunn’s multiple-comparison test were used for multiple comparisons (*P* < 0.05). A paired *t* test was used for comparisons of two groups (SMKP01 versus SMKP01*mutS*_A307T_) (*P* < 0.05).

### Data availability.

Genome sequences were deposited at the DDBJ/ENA/GenBank under accession numbers AP023148 (the SMKP03 genome), AP023149 (pSMKP03L), AP023150 (pSMKP03M), and AP023151 (pSMKP03S). Short-read genome sequence data for SMKP01, SMKP02, and SMKP03 were deposited at DDBJ under BioProject ID PRJDB9688 (https://www.ncbi.nlm.nih.gov/bioproject/?term=PRJDB9688) and BioSample IDs SAMD00220050 (SMKP01), SAMD00220051 (SMKP02), and SAMD00220052 (SMKP03).

10.1128/mBio.01954-20.4 FIG S4 Analysis of colistin-heteroresistant subpopulations of SMKP03-derived colistin-resistant mutants by PAP test. Values of 5 × 10^−1^ and 1 × 10^−7^ are highlighted by broken lines because values in this range indicate the presence of an (unstable) colistin-resistant population. Download FIG S4, PDF file, 0.1 MB. Copyright © 2020 Sato et al. 2020 Sato et al. This content is distributed under the terms of the Creative Commons Attribution 4.0 International license.
