# Supplementary figures and images for: Emergence of the Novel Aminoglycoside Acetyltransferase Variant aac(6′)-Ib-D179Y and Acquisition of Colistin Heteroresistance in Carbapenem-Resistant Klebsiella pneumoniae Due to a Disrupting Mutation in the DNA Repair Enzyme MutS
Source: mBio. 2020 Dec 22;11(6):e01954-20. doi: 10.1128/mBio.01954-20 (PMC8534291; doi:10.1128/mBio.01954-20)

Fig S1

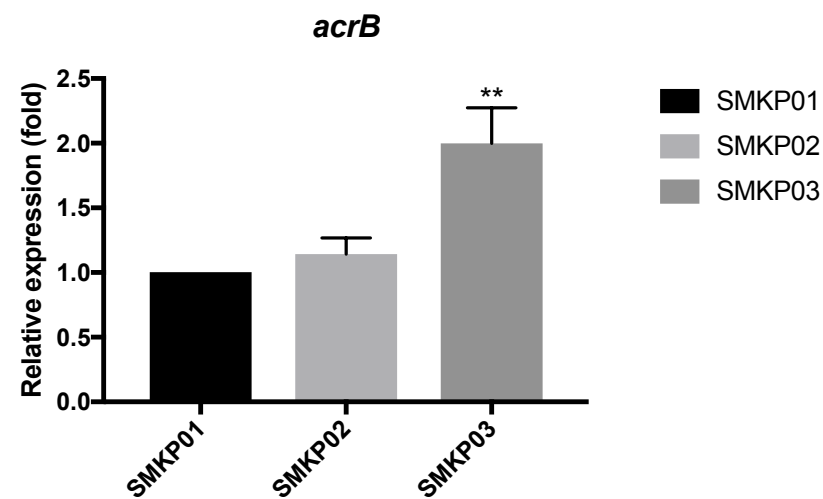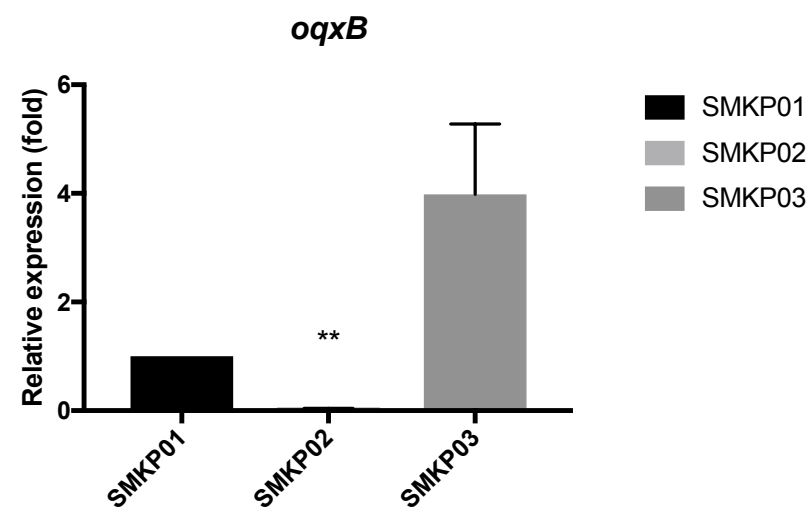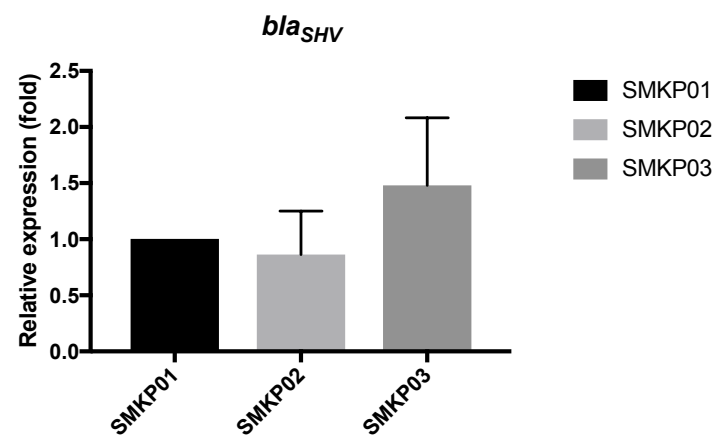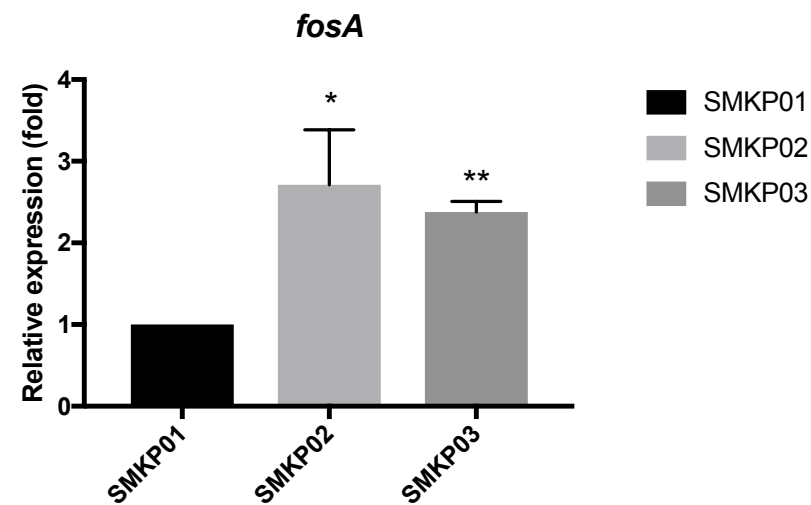

Supplement: FIG S1 [file mbio.01954-20-sf001.pdf]

Fig S2

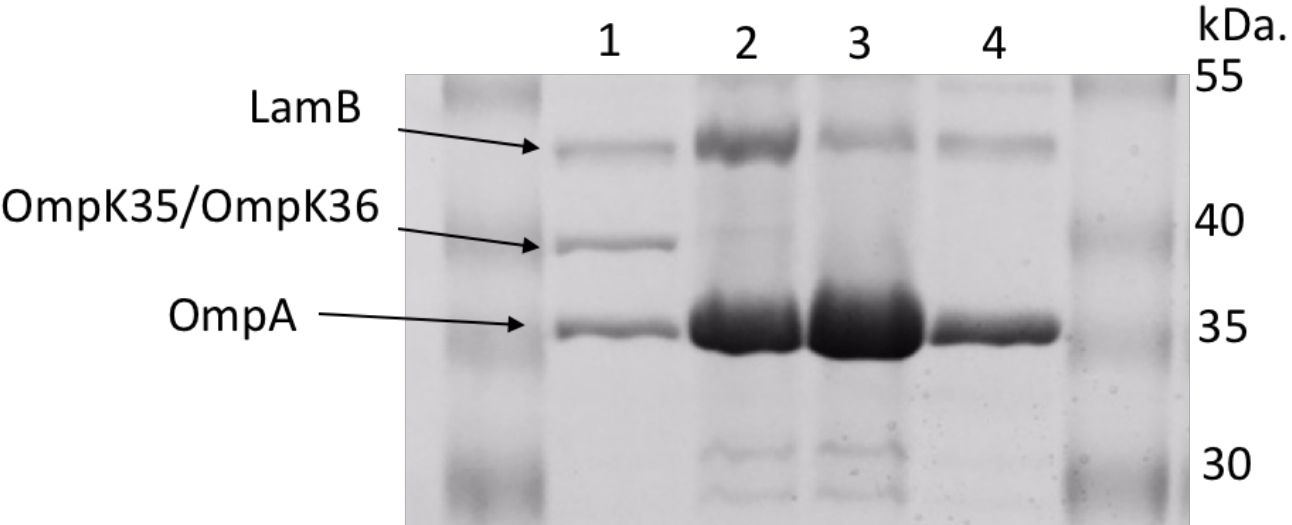

Supplement: FIG S2 [file mbio.01954-20-sf002.pdf]

Fig S3

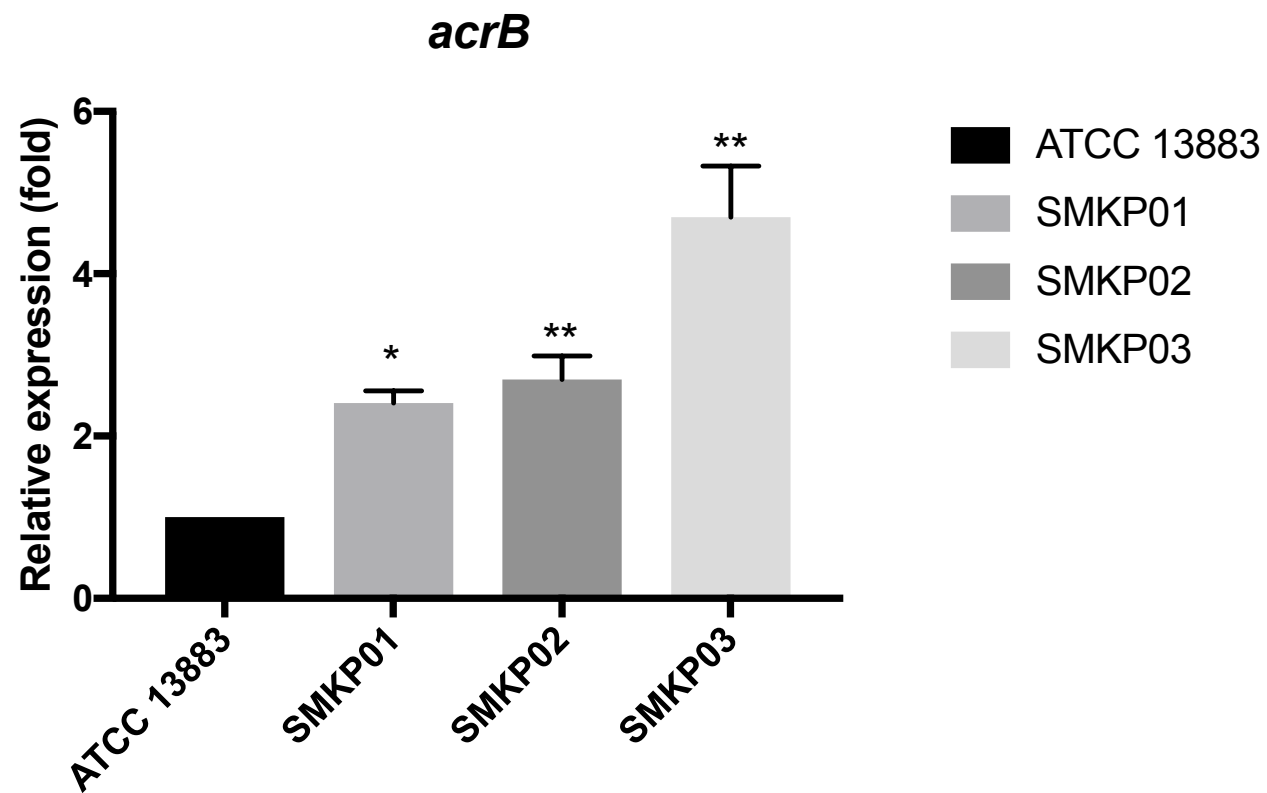

Supplement: FIG S3 [file mbio.01954-20-sf003.pdf]

Fig S4

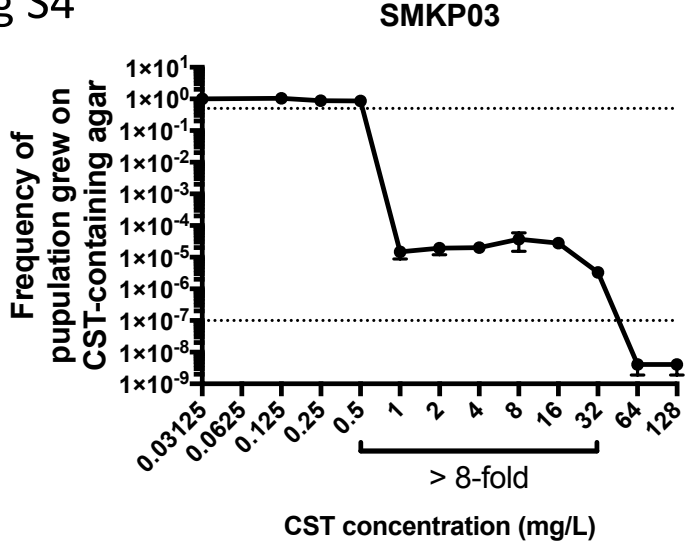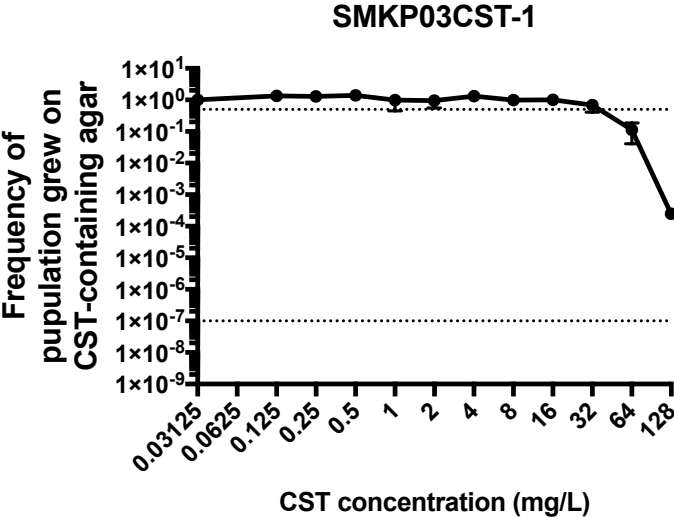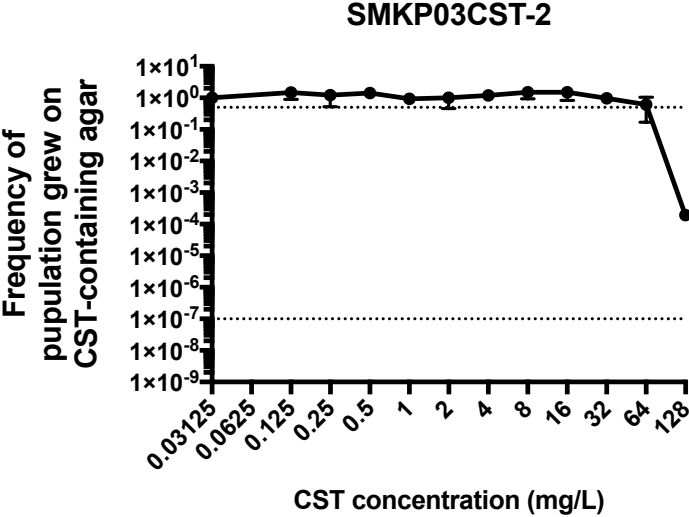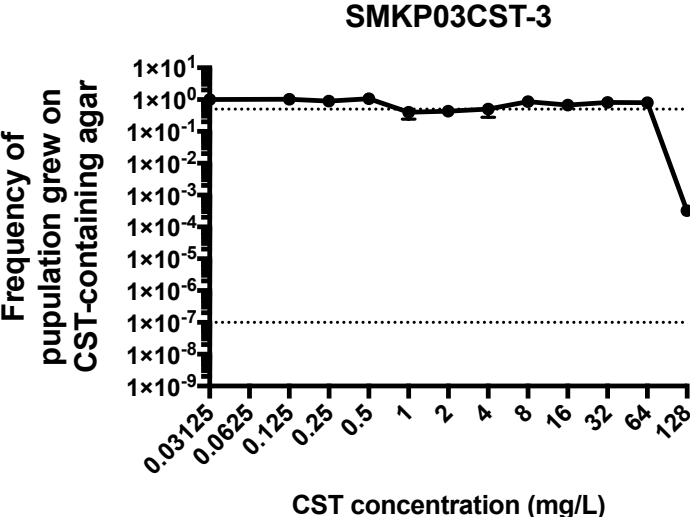

Supplement: FIG S4 [file mbio.01954-20-sf004.pdf]
